# Global Synthesis of Drought Effects on Food Legume Production

**DOI:** 10.1371/journal.pone.0127401

**Published:** 2015-06-10

**Authors:** Stefani Daryanto, Lixin Wang, Pierre-André Jacinthe

**Affiliations:** Department of Earth Sciences, Indiana University-Purdue University Indianapolis (IUPUI), Indianapolis, Indiana, United States of America; Tennessee State University, UNITED STATES

## Abstract

Food legume crops play important roles in conservation farming systems and contribute to food security in the developing world. However, in many regions of the world, their production has been adversely affected by drought. Although water scarcity is a severe abiotic constraint of legume crops productivity, it remains unclear how the effects of drought co-vary with legume species, soil texture, agroclimatic region, and drought timing. To address these uncertainties, we collected literature data between 1980 and 2014 that reported monoculture legume yield responses to drought under field conditions, and analyzed this data set using meta-analysis techniques. Our results showed that the amount of water reduction was positively related with yield reduction, but the extent of the impact varied with legume species and the phenological state during which drought occurred. Overall, lentil (*Lens culinaris*), groundnut (*Arachis hypogaea*), and pigeon pea (*Cajanus cajan*) were found to experience lower drought-induced yield reduction compared to legumes such as cowpea (*Vigna unguiculata*) and green gram (*Vigna radiate*). Yield reduction was generally greater when legumes experienced drought during their reproductive stage compared to during their vegetative stage. Legumes grown in soil with medium texture also exhibited greater yield reduction compared to those planted on soil of either coarse or fine texture. In contrast, regions and their associated climatic factors did not significantly affect legume yield reduction. In the face of changing climate, our study provides useful information for agricultural planning and research directions for development of drought-resistant legume species to improve adaptation and resilience of agricultural systems in the drought-prone regions of the world.

## Introduction

Legumes rank among humanity's most important agricultural food crops. They are grown in almost every climatic region and on a wide range of soil types. Legumes are only second to cereals in terms of contribution to food security [1], serve as major cash crop for more than 700 million smallholders in the developing countries, valued at about US$ 31 billion annually [2]. Most of that economic value comes from the export of soybean (83.8%), common bean (8.8%), groundnut (peanut) (4.9%) and chickpea (2.4%) [2]. Some legumes are grown as forages while others serve as important sources of soil nitrogen (N). Legumes have positive impacts on yield when grown in rotation or as cover crops with cereals; they have also been found to increase soil carbon (C) and N content, improve the resistance of soil to erosion, and reduce the incidence of certain soil pathogens [3, 4]. When used as manure in conservation agriculture, legumes can enhance soil porosity and reduce bulk density [5]. Promoting legume cultivation in developing countries could therefore emerge as an effective approach to achieving the Millenium Development Goals of reducing poverty and hunger, improving health and maintaining environmental sustainability [2].

World demand for legumes is expected to grow in the foreseeable future, not only in developing countries, but also in the developed nations given the trend towards healthy dieting. As the therapeutic uses of legumes are better understood [6] and the health risk of consuming animal proteins is more widely recognized, the demand for legume-based products is expected to maintain its upward trajectory. Most legumes are rich in proteins (i.e., >20%) and soluble fiber. Frequent intake of legumes has been associated with reduction in the risk of cardiovascular diseases, diabetes, digestive tract diseases, and obesity [6]. Consequently, global legume production increased from 150 million tons in the 1980’s to 300 million tons in the 2000’s. Legume production is dominated by soybean while pulses accounted for approximately 20% of total production during the same period [7]. The Food and Agricultural Organization (FAO) of the United Nations defines pulses as annual leguminous crops yielding from 1 to 12 grains or seeds of variable size, shape and color within a pod. This term is reserved for crops harvested solely for the dry grains and therefore excludes: (i) green beans and green peas, which are considered vegetable crops, as well as (ii) clover and alfalfa, which are used solely for sowing purposes (Table 1). The FAO definition also excludes soybean and groundnut from pulses since they are mainly grown for oil extraction [8]. However, this paper will include soybean and groundnut in the analyses, since they are among the top three legumes in terms of economic importance [2] and production [9].

**Table 1:** The name, origin or center of diversity [21], world production and top world producer of different types of pulses, soybean and groundnut.

No.	Pulses	Latin name	Center of origin or diversity	Production in 2013 in tons (x10^6^)	Top producers (in descending order, average of 1993–2013)
1.	Dry bean*			23.1(5.86%)**	China, India, Brazil, Myanmar
	Kidney bean, pinto bean, haricot bean, navy bean, common bean	*Phaseolus vulgaris*	Southern Mexican and Central American Center		
	Lima bean, butter bean	*Vigna lunatus*			
	Adzuki bean	*Vigna angularis*			
	Mung bean, golden gram, green gram	*Vigna radiata*	India and Pakistan		
	Black gram, urd	*Vigna mungo*	India and Pakistan		
	Scarlet runner bean	*Phaseolus coccineus*			
	Rice bean	*Vigna umbellata*			
	Moth bean	*Vigna acontifolia*			
	Tepary bean	*Phaseolus acutifolius*			
2.	Dry broad bean, horse bean, broad bean, field bean	*Vicia faba*	Central Asia Center (India, Pakistan, Afghanistan, south Russia), Middle East Center (Iran, Iraq), Mediterranean Center (Turkey, Greece, Lebanon), Africa (Ethiopia)	3.4 (0.86%)	China, Ethiopia, Egypt, Australia
3.	Dry pea, garden pea	*Pisum sativum*	Central Asia Center (India, Pakistan, Afganistan, south Russia), Middle East Center (Iran, Iraq), Mediterranean Center (Turkey, Greece, Lebanon), Africa (Ethiopia)	11.0 (2.78%)	Canada, France, Russia, China
4.	Chickpea	*Cicer arietinum*	Central Asia Center (India, Pakistan, Afganistan, south Russia), Middle East Center (Iran, Iraq), Mediterranean Center (Turkey, Greece, Lebanon), Africa (Ethiopia)	13.1 (3.32%)	India, Turkey, Pakistan, Australia, Iran
5.	Dry cowpea, blackeye pea, blackeye bean	*Vigna unguiculata*	Uncertain, but probably Indian or Ethiopian	5.7 (1.45%)	Nigeria, Niger, Burkina Faso, Tanzania, Myanmar
6.	Pigeon pea, cajan pea, congo bean	*Cajanus cajan*	Indian Center (India, Pakistan)	4.7 (1.20%)	India, Myanmar, Malawi, Tanzania, Kenya
7.	Lentil	*Lens culinaris*	Central Asia Center (India, Pakistan, Afganistan, south Russia), Middle East Center (Iran, Iraq), Mediterranean Center (Turkey, Greece, Lebanon), Africa (Ethiopia)	4.9 (1.25%)	India, Canada, Turkey, USA, Nepal
8.	Bambara bean, Bambara groundnut, earth pea	*Vigna subterranea*		0.2 (0.06%)	Burkina Faso, Mali, Niger, Cameroon, Congo
9.	Lupin	*Lupinus* spp.		0.8 (0.2%)	Australia, Belarus, Poland, Chile, Germany
10.	Vetch, common vetch	*Vicia sativa*		0.7 (0.18%)	Russia, Turkey, Ethiopia, Mexico, Spain
11.	Pulses nes			5.2 (1.32%)	India, Australia, UK, Poland, Mozambique
	Lablab bean, hyacinth bean, dolichos bean	*Dolichos lablab* or *Lablab purpureus*	Indian Center (India)		
	Jack bean, sword bean	*Canavalia ensiformis*			
	Winged bean	*Psophocarpus tetragonolobus*			
	Guar bean	*Cyamopsis tetragonoloba*			
	Velvet bean	*Stizolobium atterimum*			
	Yam bean	*Pachyrrhizus erosus*			
	**TOTAL PULSES**			73.0 (18.50%)	India, China, Canada, Brazil
	**Non-pulses**				
1.	Soybean	*Glycine max*	Chinese Center (north and central China)	276.4 (70.02%)	USA, Brazil, Argentina, China
2.	Groundnut, peanut, arachide, earthnut, monkeynut, goober pea	*Arachis hypogea*	Brazil and Paraguay Center	45.3 (11.48%)	China, India, Nigeria, USA
	**TOTAL LEGUME**			394.7	

The data are from Food and Agricultural Organization [8, 9].

*should only include *Phaseolus* spp., but some *Vigna* spp. are also included since in the past they were classified as *Phaseolus*.

**number in brackets is the percentage of total legume production.

With the expected 40% increase in world population, the agricultural sector faces an immediate challenge to increase food production by 70% or even 100% by 2050 [10, 11]. This challenge is further compounded by the severe competition for land and water from industry and urban development [12]. Such competition pushes agriculture to marginal areas, where water-limiting conditions often constrain crop productivity. Besides the persistent water limitation and year to year fluctuations of meteorological conditions in these marginal areas (e.g., semi-arid environments) tend to be large, and these variations significantly affect food security in these rain-fed systems. For example, groundnut yield in India varied between 550 and 1100 kg ha^-1^ due mainly to fluctuation in annual rainfall [13].

Droughts can negatively impact the yield of most cultivated crops, from monocotyledons C_4_ (e.g., maize) to eudicotyledons C_3_ cereals (e.g., wheat) and legumes [14–16]. The yield of food legumes grown in arid to semi-arid environments or drylands such as the Mediterranean (e.g., faba beans, chickpea and lentil), are usually variable or low due to terminal droughts that characterize these areas [17, 18]. Even in non-dryland countries like Brazil where precipitation is generally sufficient for legume (i.e., soybean) cultivation, water deficiency may still occur over a period of a few weeks, causing significant yield loss [19].

Currently, the economically viable approaches to support crop production under drought are still limited [20]. More importantly, it remains unclear how the impact of drought on legume production varies with legume species, regions, agroecosystems, soil texture, and drought timing. By synthesizing the results of field studies and drought manipulation experiments across the globe, this study aims to better characterize the factors that determine the magnitude of yield loss in legumes due to drought stress, which must be considered in agricultural planning to increase the resilience of legume production systems. The results of this study could also inform the development and selection of existing legume species, as well as better management for the drought-prone regions of the world by testing whether these species become more or less sensitive to climate variations, particularly drought. For the purpose of this study, we define drought from the agronomic point of view where there is a reduction in grain yield due to water deficit. Our main research questions are: 1) how does drought-induced legume yield reduction vary with different species, regions, climate and soil texture, and 2) what we can learn from investigating the effect of different factors and how this knowledge can help minimizing legume yield reduction in drought-affected regions?

## Methods

**Fig 1:**
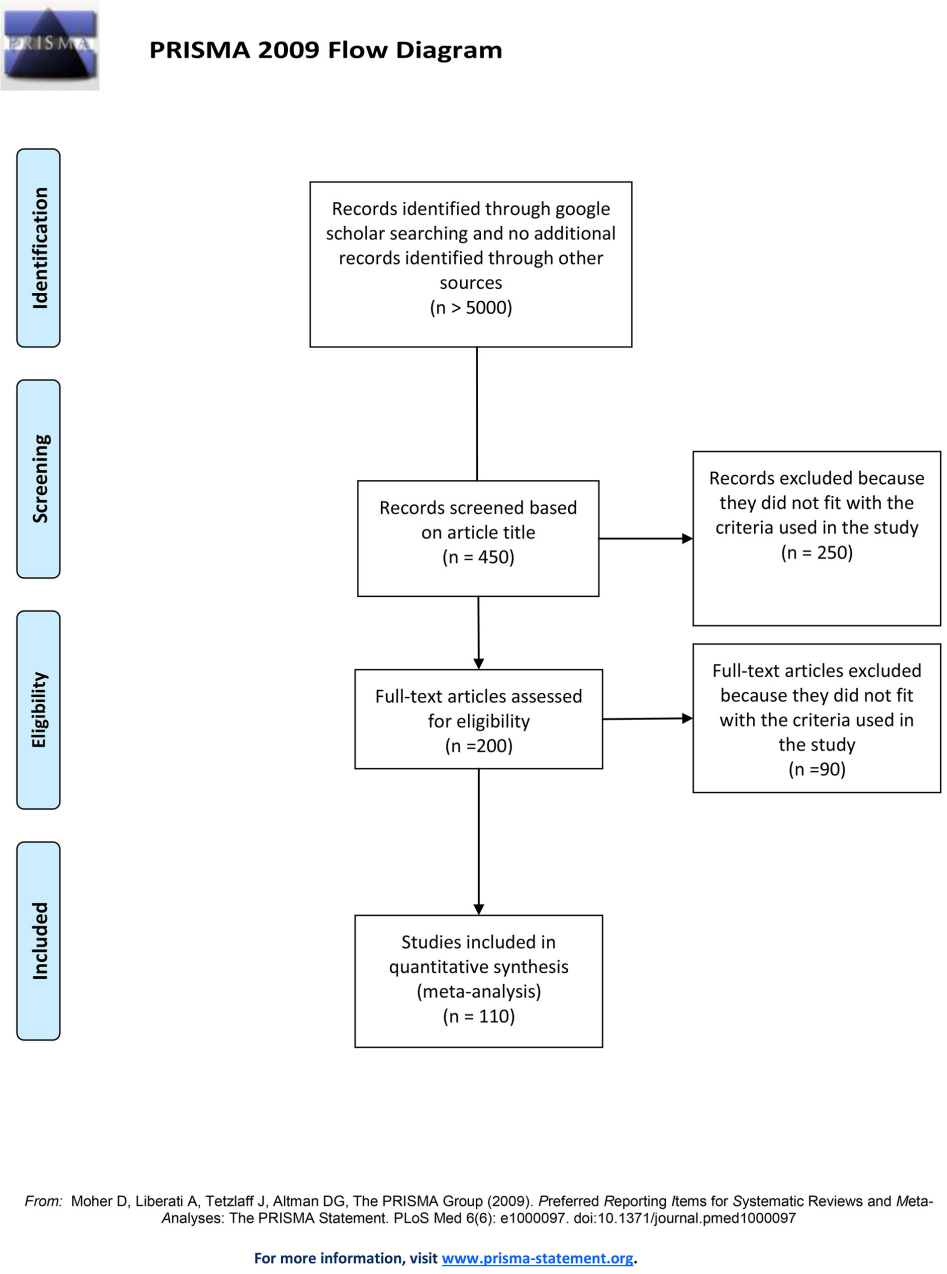
Flowchart diagram of the process of obtaining literature data to build a database for this study.

Peer-reviewed journal articles from 1980 to 2014 were collected from Google scholar using “legume species common name, water, stress, yield and field”, or “legume species common name, irrigation, deficit, yield and field” as keywords to build the database for this study. Flowchart diagram on how the process was conducted is presented in Fig 1 and the PRISMA Checklist is available via S1 File. Any article published in English during that period and meeting the following criteria was included in the database: (i) plants that experienced drought under field conditions (excluding pot studies), and the effect of water deficit was considered in comparison with well-watered condition and not in combination with other treatments (e.g., addition of fertilizers or growth hormones, modification of temperature or CO_2_), (ii) the reported plants were monoculture soybeans, groundnuts or pulses according to the FAO definition (vetch and lupin were excluded because they are mainly grown for feed) [8] (Table 1), (iii) the articles reported the response of yield per unit area. If an article presented the response of different cultivars under the same drought condition (e.g., timing), those responses were averaged across cultivars since we are interested in evaluating the effect of drought on crop performance only at the species level. However, if the same treatment was repeated over several years or locations, the data were only averaged across the years or locations if there was no significant year or location effect.

**Fig 2:**
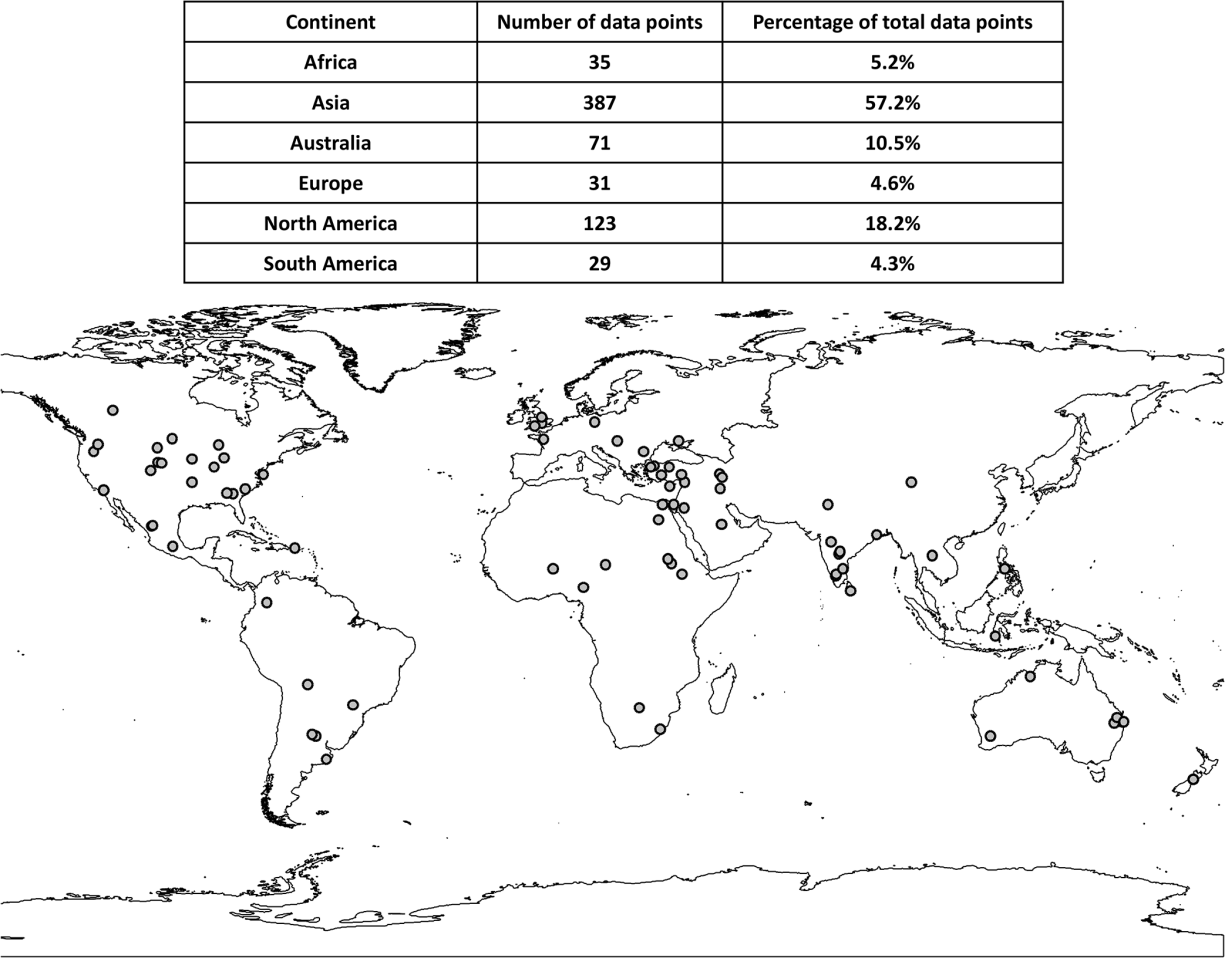
Distribution of the locations of all the studies used in this synthesis.

Distribution of the locations of studies is presented in Fig 2. Before averaging, the total number of data points was 1705 from 111 studies (S1 Table), including 325 data points where rainfed agriculture was compared with irrigated conditions. In water-limited ecosystems where rainfall is usually insufficient for crop production, rainfed agriculture usually receives less water and thus considered as “drought-affected” when compared to irrigated condition. After averaging, the number of data points was reduced to 676. We did not differentiate among irrigation types, and only recorded the amount of water applied. If a study reported more than one level of drought timing or water reduction, all observations were considered independent and included in the database.

We were particularly interested in how different categorical variables influenced the magnitude of yield responses. The categorical variables were: (i) legume species (i.e., soybean, groundnut, common bean, black gram, green gram, faba bean, field pea, chickpea, pigeon pea, cowpea, lentil, bambara bean, and lablab bean), (ii) location (i.e., tropic or non-tropic), (iii) agroecosystem type (i.e., dryland or non-dryland), (iv) drought timing (i.e., during plant’s vegetative stage, flowering or early reproductive stage, pod filling or late reproductive stage, flowering and pod filling or reproductive stage, and the entire lifespan of the plant or throughout growing period), and (v) soil texture (i.e., fine-, medium-, or coarse-textured soil). For the purposes of meta-analysis, we established discrete levels for each variable and coded each observation accordingly.

Since most of the studies were controlled experiments (i.e., comparing certain amount of irrigated conditions and drought conditions), widely-accepted drought intensity indices (e.g., Palmer index) could not be used in this study. Instead, we calculated observed water reduction (i.e., the ratio between water during drought and during well-watered condition) for each categorical variable. The exact number of data points for each category is shown in the corresponding figures since not all studies reported the amount of water reduction. One-way ANOVA was used to compare the observed water reduction under each categorical variable.

In order to include those studies that did not adequately report sample size or standard deviation, we performed an unweighted analysis using the log response ratio (lnR) to calculate bootstrapped confidence limits using the statistical software MetaWin 2.0 [22]. The difference is considered significant if the bootstrap confidence interval did not overlap with each other. A statistical significance level of *P* < 0.05 was used.

## Results and Discussion

Besides soil degradation and heat stress [2], drought is the abiotic factor that most adversely affects legume production. It turns out, however, that the largest producers of pulses (70% of global production) [7] are located in regions that experience water shortage (e.g., India, China and many African countries; Table 1) [7, 23]. These countries thus rely heavily on variable rainfall to support agriculture production which, consequently, is highly vulnerable to drought. It is also important to recognize that the impact of drought on crop yield can be variable, and therefore there is a need to consider legume crop and management factors (e.g., species selection, planting date) as these can determine crop response to water shortage and ultimately yield loss. In this study, we focused on the effect of crop species, plant phenological stage, climate, location and soil texture on yield reduction.

### Differences in species response to drought

**Fig 3:**
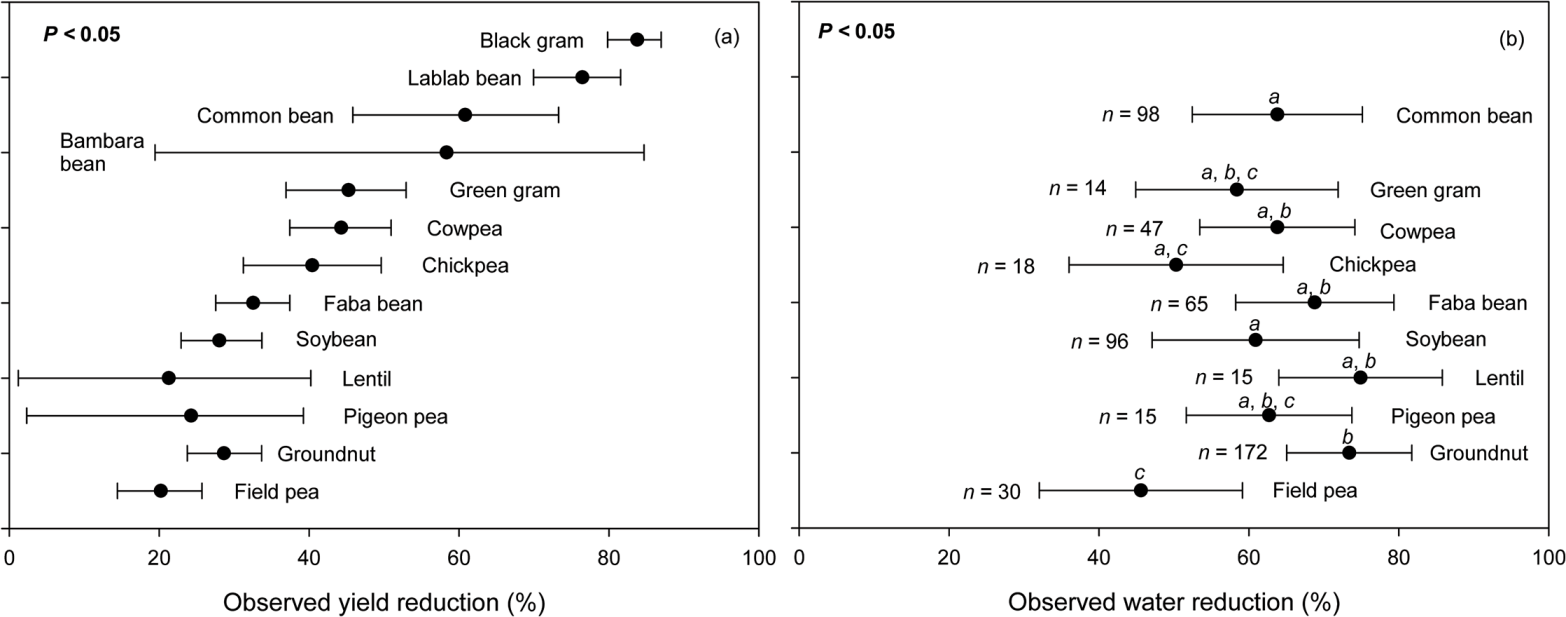
Observed yield reduction (a) and observed water reduction (b) of various legume species.

**Fig 4:**
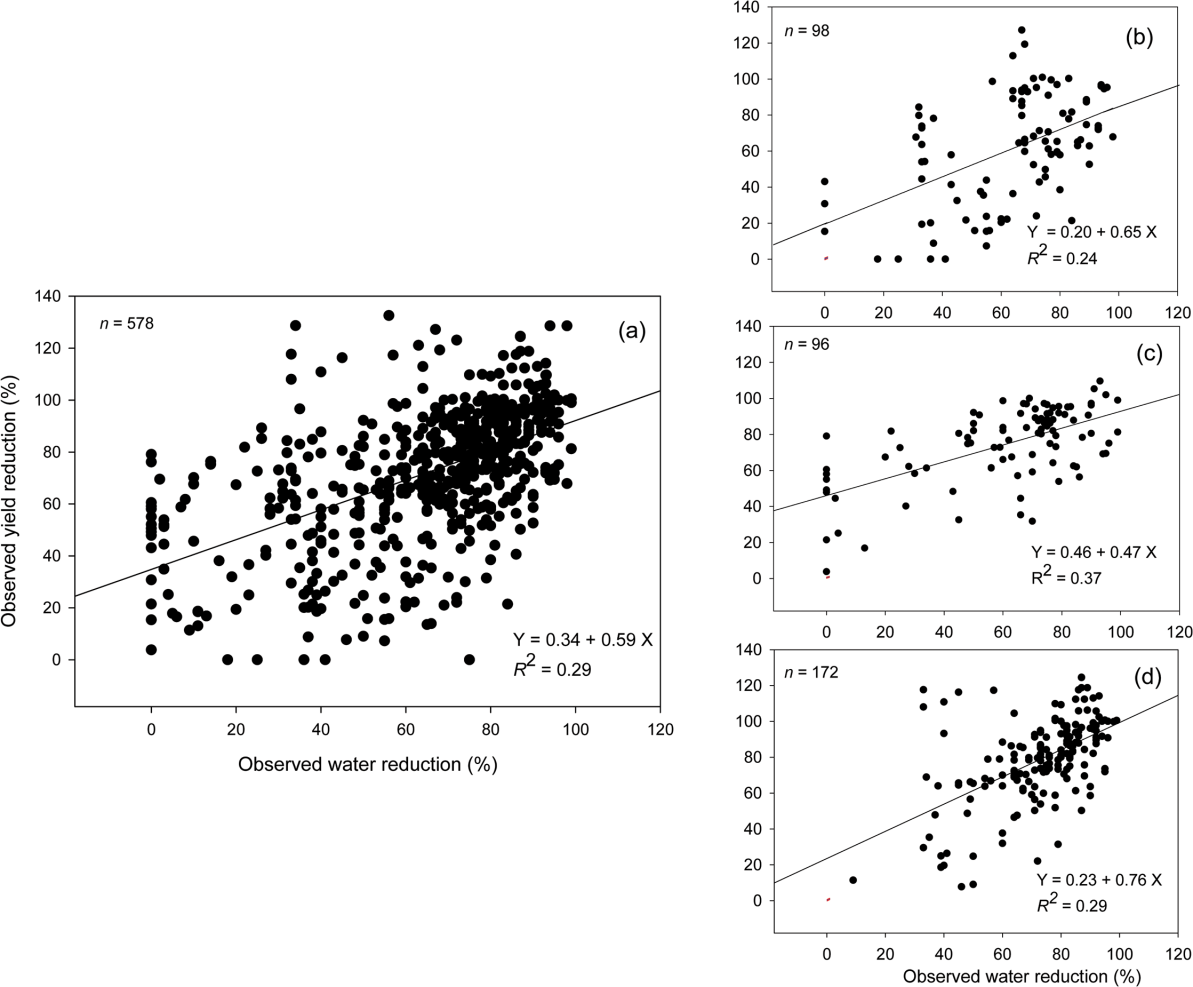
Relationship between observed yield reduction and observed water reduction of all legume species (a), common bean (b), soybean (c), and groundnut (d).

Our results showed that there were significant differences (*P* = 0.0205) among legume species with regard to their adaptability to drought as measured by their ability to maintain high yield following a period of water stress (Fig 3). Lentil and groundnut were the legumes that exhibited the lowest yield reduction (i.e., 21.7% and 28.6% for lentil and groundnut, respectively) while faba bean had the highest yield reduction (40%) under the highest observed water reduction (i.e., >65%). Under slightly lower water reduction (i.e., 60–65%), pigeon pea exhibited the lowest yield reduction (i.e., 21.8%) followed by soybean (28.0%), chickpeas (40.4%), cowpeas (44.3%), green grams (45.3%), and common beans (60.8%). Under the lowest water reduction (i.e., <60%), field pea experienced only half the amount of yield reduction observed when compared with chickpea (Fig 3). Although the amount of yield reduction varied among species, there were consistent positive linear relationships between observed yield reduction (i.e., ratio between yield during drought and during well-watered condition) and the corresponding observed water reduction across different species of legumes (Fig 4). The slopes of the regression line provide reference values for legume yield responses to drought in various regions and at the global scale. Additionally, the sensitivity of yield reduction, as indicated by the slopes of the regression lines, was different for different legume species (Fig 4B–4D).

Currently, cultivated food legumes come from different parts of the world. There are some legume crops (e.g., soybeans and common beans) that have migrated successfully from their center of origin while others (e.g., pigeon peas, green grams and black grams) remain largely confined to their areas of origin (Table 1) [21]. It has also been reported that the majority of legume crops come from dry areas while the only legume that originates from tropical regions is groundnut (Table 1) [21]. During the evolutionary history of domesticated species, the wild types generally adapt themselves to their environment of origin, ensuring their own survival and that of their progeny. At the same time, genetic variability may exist within a legume species, from extremely drought-sensitive to drought-resistant types. This dryland origin, however, does not always correspond to the adaptability of a legume species to drought. Groundnut, for example, showed a better adaptability to drought compared to common bean or black gram, even under higher level of water reduction (Fig 3). Through conventional breeding and genetic engineering, most economically valuable legume crops (e.g., soybean, groundnut) have undergone significant genetic improvement, leading to the development of varieties that are significantly more drought-resistant than their ancestors [24]. Nevertheless, there are many other legume crops that have not reached a similar level of modification. There was a variety of drought sensitivity for various legume species including the top three economic legumes (Fig 4B–4D). This indicates that most legumes may have the potential to be modified into more drought-resistant species.

Legume plants have at least two ways to resist drought: (i) drought avoidance via efficient stomata regulation, and (ii) drought tolerance via osmotic adjustment which usually allows root growth to proceed under drought condition [25–27]. Legume plants such as common bean, cowpea, and lupin are able to maintain their leaf water content and avoid tissue dehydration during light drought by controlling their stomatal conductance and closure [28–30]. Such closure, consequently, can lead to a decrease in internal CO_2_ concentrations, which eventually limit photosynthesis and shoot growth. On the other hand, the second mechanism—osmotic regulation through increased solute concentration—is less energy demanding [31]. Therefore, this second mechanism less severely impacts productivity than the first [32]. The solutes, which mostly consist of organic substrates (i.e., sugars, sugar alcohols and amino compounds), are allocated to the roots to lower their osmotic potentials [33–35]. This mechanism allows the roots to continue extracting water at low soil water potentials [36]. Maintaining turgor and plant water content by lowering epidermal conductance have been an important trait for several legumes (e.g., chickpea, cowpea, common bean, pigeon pea) [31, 37], while lowering osmotic potential has been observed in other beans (e.g., common bean, faba bean and cowpea) in response to water deficit. Some legumes may use both mechanisms (e.g., common bean and cowpea) while other species (e.g., chickpea) can only use one mechanism [31]. The use of the more energy-demanding mechanism or even the use of both mechanisms, however, did not always translate into lower yield reduction (Fig 3), most likely because the mechanisms interact with other physiological factors such as N-fixing trait.

Legumes are unique in their capacity to resist drought because of their interaction with N-fixing (i.e., rhizobia) bacteria and arbuscular mycorrhiza [38–40]. Although some studies have suggested that N_2_ fixation might be inhibited by water deficit [41], numerous lines of evidence have shown that genetic variation exists among species and that may be responsible for their variable resistance to water stress [42, 43]. This N-fixing trait could be an important determining factor of yield potential [44] since legume plants need to combine biomass accumulated from photosynthesis with fixed N to form the essential components of the grain [45]. Variety in nodule typology could also be responsible for the higher N_2_ fixation of some legumes. For example, nodules formed at the endodermis (i.e., indeterminate nodules) such as in faba bean and groundnut, are able to resist water stress better than those that are superficially attached (i.e., determinate nodules) such as in cowpea, black gram and green gram [46]. While indeterminate nodules are able to grow rapidly after periods of adverse conditions, the determinate nodules are short-lived and must be replaced when plant growth resumes. This replacement process can sometimes be incomplete [46], limiting the efficiency of N_2_ fixation. Consistent with our findings, the legumes species that exhibit relatively high N_2_ fixation during drought (e.g., groundnut and faba bean) also tend to produce higher yields during drought compared to the species that have limited N_2_ fixation during drought (e.g., green gram, black gram and cowpea) [41]. Some legume species thus may benefit more from the symbiosis than others since the investment to maintain the nodules is about the same for most plants (i.e., about 20% of net photosynthate) [47]. Therefore, species might be selected for less sensitivity of N_2_ fixation to water deficits in regions where drought is a recurring phenomenon.

### Differences in drought responses under different plant phenological stages

**Fig 5:**
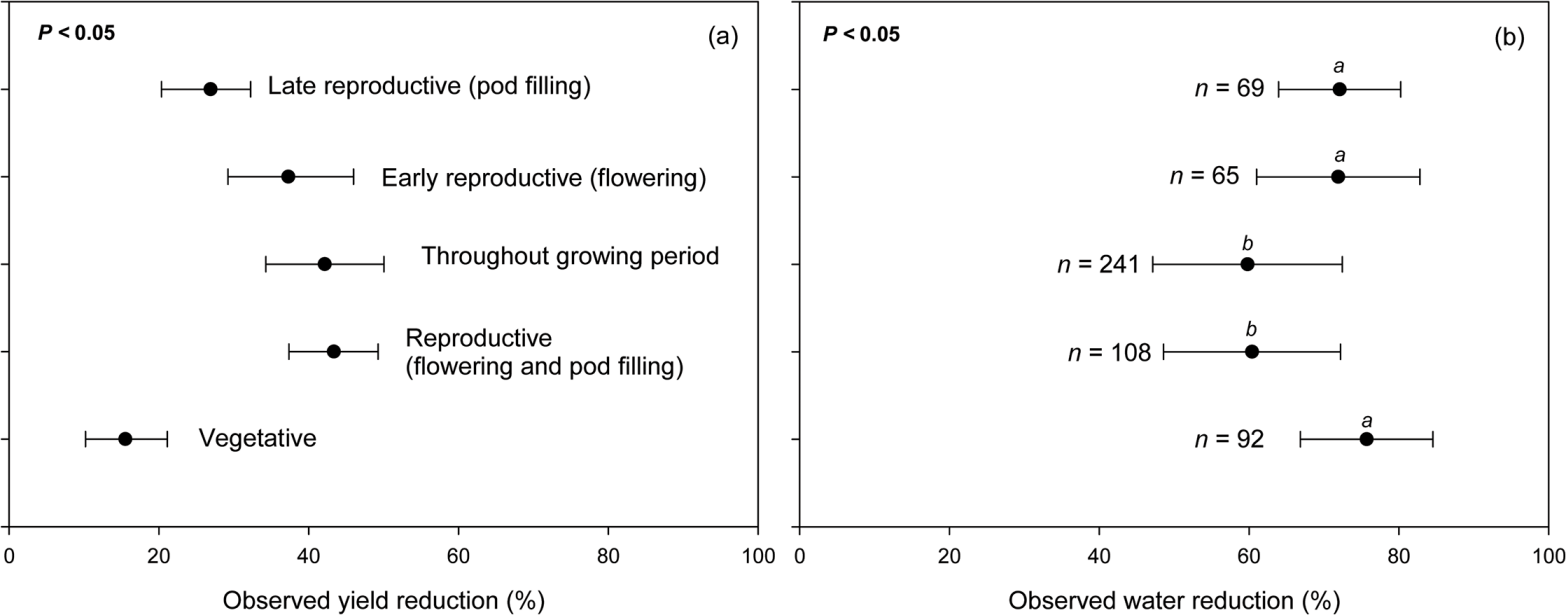
Observed yield reduction (a) and observed water reduction (b) of various legume species at different phenological stages.

Plant phenological stage affected the percentage of yield reduction observed in legume crops, with drought during the vegetative phase resulting in lowest yield reduction (15.5%; *P*<0.01) compared to drought that occurred during the early and late reproductive stages under the same amount of water reduction (Fig 5). Although drought during the very early vegetative stage may impair germination, most studies that examined the effect of drought usually allowed sufficient water to support good and uniform plant establishment. Therefore, drought that happens during the later vegetative periods (e.g., trifoliate formation) was relatively more tolerable to plants even though they might experience retarded cell elongation, division and differentiation [48]. They are still able to maintain their growth functions under stress because early drought may lead to immediate survival or acclimation where the plants modify their metabolic and structural capabilities mediated by altered gene expression [27].

The reproductive stage is often the most critical phase influencing the yield of crops harvested for grains or seeds. Our results showed that drought that occurred during reproductive stages (i.e., from flowering to maturity) resulted in yield reduction (43.4%) similar to the reduction observed when drought occurred throughout the growing season (42.1%; *P*<0.01; Fig 5). Drought usually reduces yield by one or the combination of the following mechanisms: (i) shortening the duration of reproductive development, (ii) reducing branching and consequently the number of pods [49, 50], and (iii) reducing seed weight and the number of seeds per pod [51]. Droughts that occurred during the early reproductive stage (i.e., flowering) were more devastating compared to those that occurred during the late generative stage (i.e., pod filling to maturity). Yield reduction averaged 37.3% and 26.89% for droughts that occurred during the early and late reproductive stages, respectively (Fig 5A). Droughts during the flowering stage often resulted in bareness due to a reduction in the flux of assimilate to the developing seeds. Similarly, reduction in the assimilate partitioning and activity of starch-synthesis enzymes (i.e., sucrose synthase, adenosine diphosphate glucose pyrophosphorylase, starch synthase and starch branching enzyme) occurred during the grain-filling period [48].

### Soil texture effect on drought impacts

**Fig 6:**
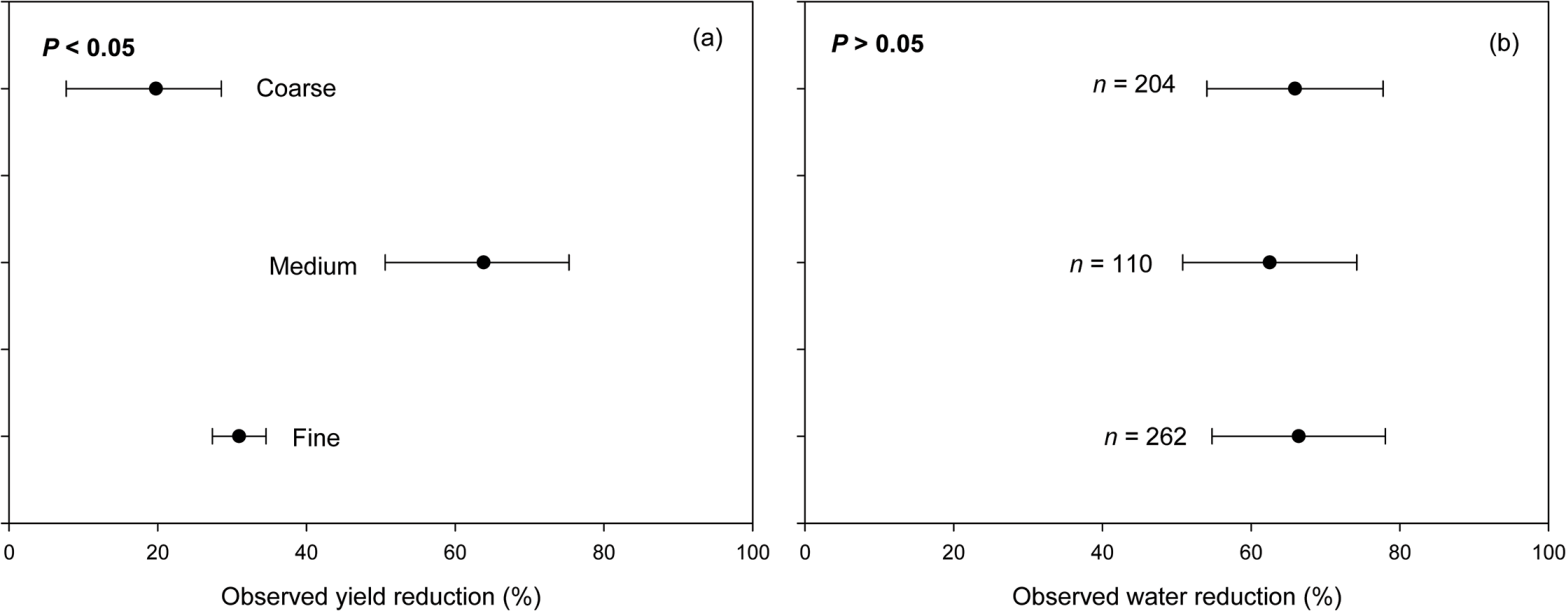
Observed yield reduction (a) and observed water reduction (b) of various legume species grown at sites of different soil textures.

We found that droughts resulted in greatest yield reduction (63.8%) in medium-textured soils compared to either fine-textured (30.9%) or coarse-textured soils (19.8%; *P*<0.001; Fig 6). This pattern could be related to the potential production capacity of these soils. The inherently low natural fertility of sandy soils usually leads to lower yield potential, meaning that without significant inputs high yields are less likely even with adequate rainfall [52]. In contrast, the production potential of medium- to fine-textured soils is usually higher. Given the natural fertility of these soils [52], they provide more favorable growing condition for most legume crops when water is available [53]. However, because water in retained at much lower water potentials in fine-textured (e.g., clayed, clay-loam) than medium-textured soils, water extraction by plant roots is more difficult in clay-rich soil even under conditions of moderate soil moisture deficit [54]. Critical soil water potential (i.e., below which a significant decrease of water extraction can be observed) [55], however, is determined not only by soil texture but also by the type of plants, particularly the trait related to root density [54]. While greater root growth normally supports larger extraction of soil moisture, this trait is of limited importance under soil conditions that restrict root growth (e.g., in dry clayed soil) [56]. Water uptake rate of faba bean, for example, was found to be proportional to root length density at high soil water potentials, but not at potentials lower than −25 kPa [54]. In other words, root density remains an important factor in determining water uptake at certain soil water potential, although the relationship is not necessarily linear.

### Agroclimatic-region influence on drought impacts

**Fig 7:**
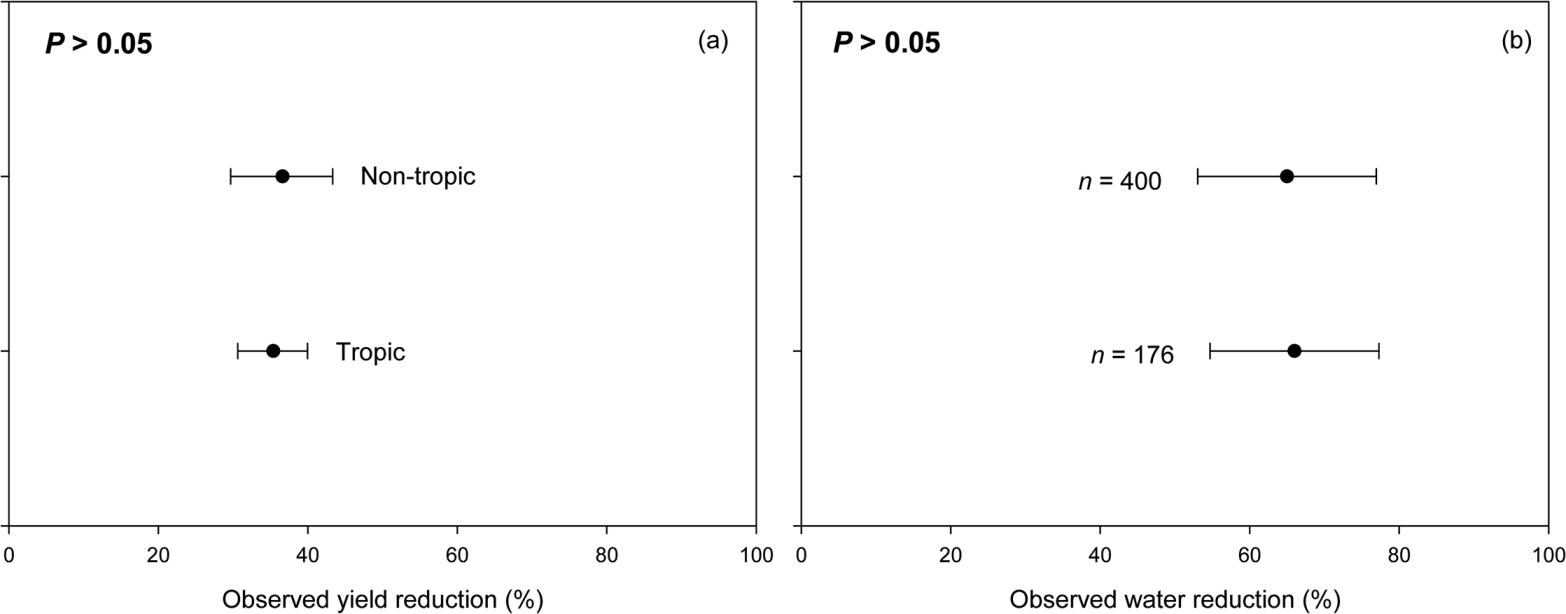
Observed yield reduction (a) and observed water reduction (b) of various legume species grown in tropical and non-tropical regions.

When separated into tropical and non-tropical regions, our results showed that no difference in yield reduction between legumes planted in the tropics (35.4%) and in the non-tropical regions (36.6%; Fig 7). However, these results need to be interpreted with caution since they were based largely on studies conducted at experimental sites where agricultural input (e.g., pest control and fertilizers) was not a limiting factor. In reality, there was significant difference between pulse productivity in the developed countries which are located mostly in non-tropical regions (1.8 tons ha^-1^) and the developing countries (0.8 tons ha^-1^) which are located mostly in tropical regions [7]. Possible reasons for these results include: 1) farmers in the tropics usually experience greater production loss due to the lack of capital and technology to support vigorous plant establishment and growth [7]; 2) soils in the humid tropics are commonly leached, highly weathered and low both in total and plant-available N, requiring high fertilizer input [57]; and 3) rapid land degradation occurs because of intensive cultivation, short fallow periods in traditional farming systems, overgrazing and tree harvesting to meet fuelwood demand of growing populations [58]. Farmers in the tropics are also dominated by aging and poorly-educated smallholders who depend very much on manual labor [2]. In the non-tropical regions, however, farming is facilitated by the availability of mechanized equipment, and farmers’ access to reliable weather forecast.

**Fig 8:**
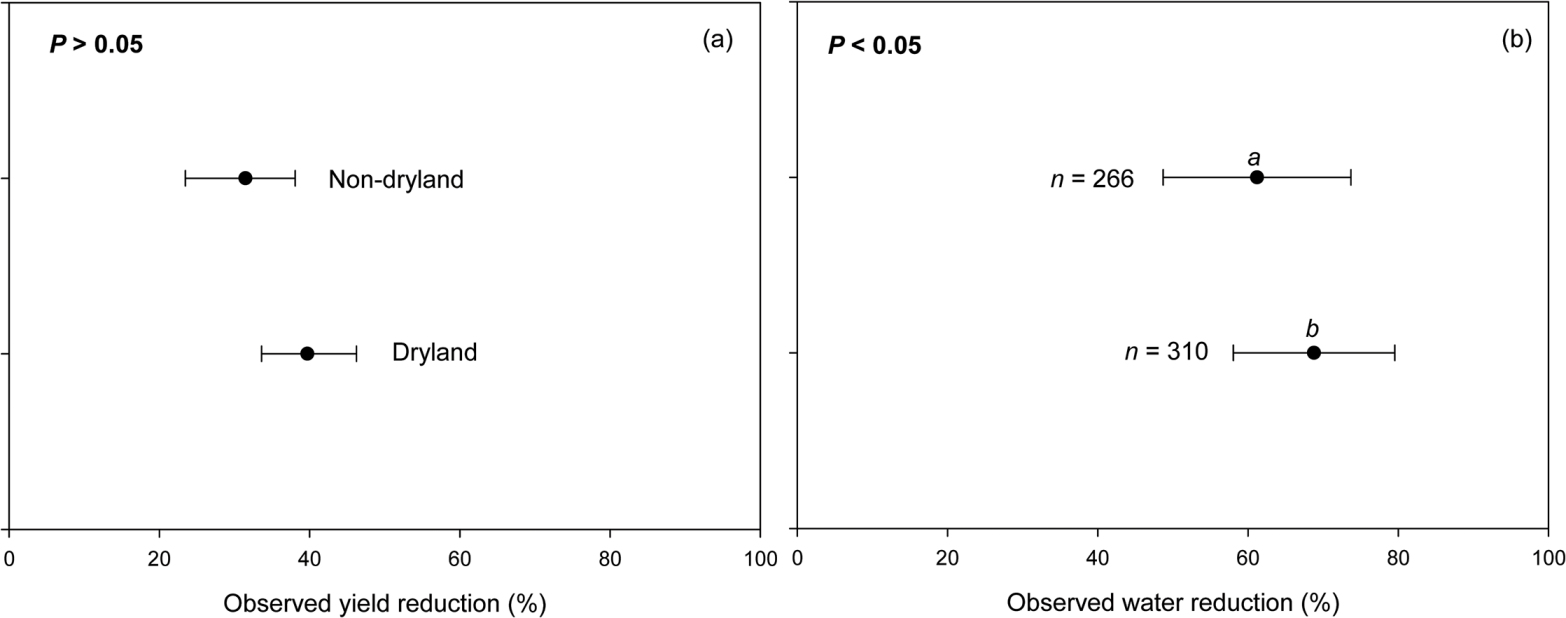
Observed yield reduction (a) and observed water reduction (b) of various legume species grown in dryland and non-dryland regions.

We did not find any significant difference in crop yield between legumes grown in dryland and non-dryland regions probably because even in their natural conditions, legumes of dryland origin (e.g., chickpea, pigeonpea) usually experience terminal drought and show a yield increase if irrigation is applied during the reproductive phase [46]. The similarity of the responses to drought of legumes planted in dryland and in non-dryland areas (Fig 8) is therefore not surprising even if there is species selection and the majority of legumes originated from dry regions (Table 1) [21].

## Conclusions

In this study, through meta-analytic techniques, we investigated how the effects of drought on yield of legume crops co-vary with legume species, soil texture, agroclimatic regions, and drought timing. Many regions of the world have experienced significant shifts in the pattern and amount of rainfall, thus raising concern of a growing water scarcity problem and increasing frequency of crop failure. This study provides useful information that could inform agricultural planning and management to minimize drought-induced yield loss. Since our results showed that the effects of drought on yield reduction varied with species, soil texture, as well as drought timing, this study underscores the need to prioritize the selection and development of drought resistant legume species adapted to the drought-prone regions of the world. Since the effects of drought on legume production was found to be less affected by climatic regions (e.g., non-tropical vs. tropics or drylands vs. non-drylands) but was more related to legume species, the selection and promotion of drought-resistant legume species could provide an approach to minimize the impact of droughts. When selecting for drought-resistant species, phenological plasticity could be an important trait to consider given the irregularity in rainfall pattern and the observation that drought generally causes higher yield reduction when it occurs during the reproductive stage compared to during vegetative growth. Among the species of crop legumes, common bean could be the species that requires the most research since it ranks third among legumes in terms of production (Table 1) and second in world trade export [2], yet it exhibits high sensitivity to drought and low productivity.

## Supporting Information

S1 File PRISMA 2009 checklist. (PDF)

S1 Table List of references used to build the database of this study. (DOCX)
