# Supplementary material for: Global Synthesis of Drought Effects on Food Legume Production
Source: PLoS One. 2015 Jun 10;10(6):e0127401. doi: 10.1371/journal.pone.0127401 (PMC4464651; doi:10.1371/journal.pone.0127401)
Supplement: S1 Table — (DOCX) [file pone.0127401.s002.docx]

**S1 Table. List of references used to collect database for this study**

| **No.** | **Authors and article title** | **Journal name and edition** |
| --- | --- | --- |
| 1. | Acosta-Gallegos JA, Adams MW. Plant traits and yield stability of dry bean (*Phaseolus vulgaris*) cultivars under drought stress. | J Agr Sci. 1991; 117: 213-219. |
| 2. | Acosta-Gallegos JA, Shibata JK. Effect of water stress on growth and yield on indeterminate dry bean (*Phaseolus vulgaris*) cultivars | Field Crop Res. 1989; 20: 81-93. |
| 3. | Ahmed FE, Suliman ASH. Effect of water stress applied at different stages of growth on seed yield and water-use efficiency of cowpea. | Agric Bio J N Am. 2010; 1: 534-540. |
| 4. | Al-Kaisi MM, Berrada AF, Stack MW. Dry bean yield response to different irrigation rates in southwestern Colorado. | J Prod Agric. 1999; 12: 422-427. |
| 5. | Al-Suhaibani NA. Influence of early water deficit on seed yield and quality of faba bean under arid environment of Saudi Arabia. | Am-Eurasian J Agric Environ Sci. 2009; 5: 649-654. |
| 6. | Amede T, Kittlitz EV, Schubert S. Differential drought responses of faba bean (*Vicia faba* L.) inbred lines. | J Agron Crop Sci. 1999; 183: 35-45. |
| 7. | Andriani JM, Andrade FH, Suero EE, Dardanelli JL. Water deficits during reproductive growth of soybeans. 1 Their effects on dry matter accumulation, seed yield and its components. | Agronomie 1999; 11, 737-746. |
| 8. | Barrios AN, Hoogenboom G, Nesmith DS. Drought stress and the distribution of vegetative and reproductive traits of a bean cultivar. | Sci Agric. 2005; 62 (1), 18-22. |
| 9. | Brown EA, Caviness CE, Brown DA. Response of selected soybean cultivars to soil moisture deficit. | Agron J. 1985; 77: 274-278. |
| 10. | Chapman SC, Ludlow MM, Blamey FPC, Fischer KS. Effect of drought during pod filling on utilization of water and growth of cultivars of groundnut (*Arachis hypogaea* L.). | Field Crop Res. 1993; 32: 243-255. |
| 11. | Chapman SC, Ludlow MM, Blamey FPC, Fischer KS. Effect of drought during early reproductive development on growth cultivars of groundnut (*Arachis hypogaea* L.): 2. Biomass production, pod development and yield. | Field Crop Res. 1993b; 32: 211-225. |
| 12. | Clavel D, Drame NK, Roy-Macauley H, Braconnier S, Laffray D. Analysis of early responses to drought associated with field drought adaptation in four Sahelian groundnut (*Arachis hypogaea* L.) cultivars. | Environ Exp Bot. 2005; 54: 219-230. |
| 13. | Collino DJ, Dardanelli JR, Sereno R, Racca RW. Physiological response of argentine peanut varieties to water stress: Water uptake and water use efficiency. | Field Crop Res. 2000; 68: 133-142. |
| 14. | Cox WJ, Jolliff GD. Growth and yield of sunflower and soybean under soil water deficits. | Agron J. 1986; 78: 226-230. |
| 15. | Dadson RB, Hashem FM, Javaid I, Joshi J, Allen AL, et al. Effect of water stress on the yield of cowpea (*Vigna unguiculata* L. Walp) genotypes in the Delmarva region of United States. | J Agron Crop Sci. 2005; 191: 210-217. |
| 16. | Dapaah HK, McKenzie BA, Hill GD. Effects of irrigation and sowing date on phenology and yield of pinto beans (*Phaseolus vulgaris* L.) in Canterbury, New Zealand. | New Zeal J Crop Hort. 1999; 27: 297-305. |
| 17. | Dapaah HK, McKenzie BA, Hill GD. Effects of sowing date and irrigation on the growth and yield of pinto beans (*Phaseolus vulgaris* L.) in a subhumid temperate environment. | J Agr Sci. 1999; 134: 33-43. |
| 18. | De Costa WAJM, Shanmugathasan KN, Joseph KDSM. Physiology of yield determination of mungbean (*Vigna radiata* (L.) Wilczek) under various irrigation regimes in the dry and intermediate zones of Sri Lanka. | Field Crop Res. 1999; 61: 1-12. |
| 19. | Demirtaş C, Yazgan S, Candogan BN, Sincik M, Büyükcangaz H, et al. Quality and yield response of soybean (*Glycine max* L. Merill) to drought stress in sub-humid environment. | Afr J Biotechnol. 2010; 9: 6873-6881. |
| 20. | Dogan E, Kirnak H, Copur O. Deficit irrigation during soybean reproductive stages and CROPGRO-soybean simulations under semi-arid climatic conditions. | Field Crop Res. 2007; 103, 154-159. |
| 21. | Erickson PI, Ketring DL. Evaluation of peanut genotypes for resistance to water stress in situ. | Crop Sci. 1985; 25: 870-876. |
| 22. | Haro RJ, Dardanelli JL, Otegui ME, Collino DJ. Seed yield determination of peanut crops under water deficit: Soil strength effects on pod set, the source-sink ratio and radiation use efficiency. | Field Crop Res. 2008; 109, 24-33. |
| 23. | Ishag HM, Fadl OA, Adam HS, Osman AK. Growth and water relations of groundnut (*Arachis hypogaea*) in two contrasting years in the irrigated gezira. | Exp Agric. 1985; 21: 403-408. |
| 24. | Farah SM. An examination of the effect of water stress on leaf growth of crops of field beans (*Vicia faba* L.). | J Agr Sci. 1981; 96: 327-336. |
| 25. | Fapohunda HO, Aina PO, Hossain MM. Water use – yield relation for cowpea and maize. | Agric Water Manage. 1984; 9: 219-224. |
| 26. | Foroud N, Mündel HH, Saindon G, Entz T. Effect of timing and level of moisture stress on soybean yield, protein, and oil responses. | Field Crop Res. 1993; 31: 195-209. |
| 27. | Foroud N, Mündel HH, Saindon G, Entz T. Effect of level and timing of moisture stress on soybean plant development and yield components. | Irrig Sci. 1993; 13: 149-155. |
| 28. | Fougereux JA, Doré T, Ladonne F, Fleury A. Water stress during reproductive stages affects seed quality and yield of pea (*Pisum sativum* L.). | Crop Sci. 1997; 37: 1247-1252. |
| 29. | Frederick JR, Camp CR, Bauer PJ. Drought stress effects on branch and mainstem seed yield and yield components of determinate soybean. | Crop Sci. 2001; 41: 759-763. |
| 30. | Frederick JR, Woolley JT, Hesketh JD, Peters BD. Seed yield and agronomic traits of old and modern soybean cultivars under irrigation and soil water deficit. | Field Crop Res. 1991; 27: 71-82. |
| 31. | Garcia y Garcia A, Persson T, Guerra LC, Hoongenboom G. Response of soybean genotypes to different irrigation regimes in a humid region of southwestern USA. | Agric Water Manage. 2010; 97: 981-897. |
| 32. | Ghassemi-Golezani K, Ghassemi S, Bandehhagh A. Effects of water supply on field performance of chickpea (*Cicer arietinum* L.) cultivars. | Int J Agron Plant Prod. 2013; 4: 94-97. |
| 33. | Ghassemi-Golezani K, Lotfi R. Response of soybean cultivars to water stress at reproductive stages. | Int J Plant Anim Environ Sci. 2012; 2: 198-202. |
| 34. | Ghassemi-Golezani K, Zafarani-Moatar P, Raey Y, Mohammadi A. Response of pinto bean cultivars to water deficit at reproductive stages. | J Food Agric Environ. 2010; 8: 801-804. |
| 35. | Gohari AA. Effects of water infiltration to soil in increasing yield and water-use efficiency in peanut (*Arachis hypogaea* L.). | Am-Eurasian J Agric Environ Sci. 2011; 10: 797-801. |
| 36. | Grantz DA, Hall AE. Earliness of an indeterminate crop, *Vigna unguicolata* (L.) Walp., as affected by drought, temperature and plant density. | Aust J Agric Res. 1982; 33: 531-540. |
| 37. | Greenberg DC, Williams JH, Ndunguru BJ. Differences in yield determining processes of groundnut (*Arachis hypogaea* L.) genotypes in varied drought environments. | Ann Appl Biol. 1992; 120, 557-566. |
| 38. | Gunton JL, Evenson JP. Moisture stress in navy beans: 1. Effect of withholding irrigation at different phonological stages on growth and yield. | Irrig Sci. 1980; 2: 49-58. |
| 39. | Hamidou F, Ratnakumar P, Halilou O, Mponda O, Kapewa T, et al. Selection of intermittent drought tolerant lines across years and locations in the reference collection of groundnut (*Arachis hypogaea* L.). | Field Crop Res. 2012; 126: 189-199. |
| 40. | Işik M, Ӧnceler Z, Ḉakir S, Altay F. Effects of different irrigation regimes on the yield and yield components of dry bean (*Phaseolus vulgaris*). | Acta Agron Hung. 2004; 52: 381-389. |
| 41. | Jongrungklang N, Toomsan B, Vorasoot N, Jogloy S, Kesmala T et al. Identification of peanut genotypes with higher water use efficiency under drought stress conditions from peanut germplasm of diverse origins. | Asian J Plant Sci. 2008; 7: 628-638. |
| 42. | Jongrungklang N, Toomsan B, Vorasoot N, Jogloy S, Boote KJ et al. Rooting traits of peanut genotypes with different yield responses to pre-flowering drought stress. | Field Crop Res. 2011; 120: 262-270. |
| 43. | Karamanos AJ. Effects of water stress on some growth parameters and yields of field bean crops. | World Crop. 1984; 10: 47-59. |
| 44. | Karrou M, Oweis T. Water and land productivities of wheat and food legumes with deficit supplemental irrigation in a Mediterranean environment. | Agric Water Manage. 2012; 107: 94-103. |
| 45. | Khamssi NN, Ghassemi-Golezani K, Najaphy A, Zehtab S. Evaluation of grain filling rate, effective grain filling period and resistance indices under acclimation to gradual water deficit stress in chickpea cultivars. | Aust J Crop Sci. 2011; 5: 1044-1049. |
| 46. | Kheira AAA. Macromanagement of deficit-irrigated peanut with sprinkler irrigation. | Agric Water Manage. 2009; 96: 1409-1420. |
| 47. | Kingra PK, Kaur P. Yield and water use efficiency of groundnut (*Arachis hypogaea* L.) cultivars as influenced by time and of sowing under irrigated and rainfed conditions in central Punjab. | J Res Punjab Agric Univ. 2011; 48: 119-123. |
| 48. | Knott CM. Irrigation of spring field beans (*Vicia faba*): response to timing at different crop stages. | J Agr Sci. 1999; 132: 407-415. |
| 49. | Al-Naeem M. Effect of irrigation scheduling on growth parameter and water use efficiency of barley and faba bean crops in Al-Ahsa, Saudi Arabia. | Am J Plant Physiol. 2008; 3: 111-120. |
| 50. | Korte LL, Williams JH, Specht JE, Sorensen RE. Irrigation of soybean genotypes during reproductive ontogeny: 1. Agronomic responses. | Crop Sci. 1983; 23: 521-527. |
| 51. | Labanauskas CK, Shouse P, Stolzy LH. Effects of water stress at various growth stages on seed yield and nutrient concentrations on field-grown cowpeas. | Soil Sci. 1981; 131: 249-256. |
| 52. | Leport l, Turner NC, French RJ, Barr MD, Duda R, et al. Physiological responses of chickpea genotypes to terminal drought in a Mediterranean-type environment. | Eur J Agron. 1999; 11: 279-291. |
| 53. | Lizana C, Wenworth M, Martinez JP, Villegas D, Meneses R, et al. Differential adaptation of two varieties of common bean to abiotic stress, 1. Effect of drought on yield and photosynthesis. | J Exp Bot. 2006; 57: 685-697. |
| 54. | Lopez FB, Johansen C, Chauhan YS. Effects of timing of drought stress on phenology, yield, and yield components of short-duration pigeonpea. | J Agron Crop Sci. 1996; 177: 311-320. |
| 55. | Lyon DJ, Boa F, Arkerbauer TJ. Water-yield relations of several spring-planted dryland crops following winter wheat. | J Prod Agric. 1995; 8: 281-286. |
| 56. | Mabhaudhi T, Modi AT, Beletse YG. Growth, phonological and yield responses of Bambara groundnut (*Vigna subterranea* L. Verdc) landrace to imposed water stress: 2. Rainshelter conditions. | Agric Water Manage. 2013; 121:102-112. |
| 57. | Mafakheri A, Siosemardeh A, Bahramejad B, Struik PC, Sohrabi E. Effect of drought stress on yield, proline, and chlorophyll contents in three chickpea cultivars. | Aust J Crop Sci. 2010; 4: 58-585. |
| 58. | Martin RJ, Jamieson PD. Effect of timing and intensity of drought on the growth and yield of field peas (*Pisum sativum* L.). | New Zeal J Crop Hort. 1996; 24: 167-174. |
| 59. | McKenzie BA, Hill GD. Growth, yield and water use of lentils (*Lens culinaris*) in Canterbury, New Zealand. | J Agr Sci. 1990; 114: 309-320. |
| 60. | Metochis C. Irrigation of groundnut (*Arachis hypogaea*) grown in a Mediterranean environment. | J Agr Sci. 1993; 121: 343-346. |
| 61. | Miller DA, Burke DW. Response of dry beans to daily deficit sprinkler irrigation. | Agron J. 1983; 75: 775-778. |
| 62. | Mohamed F, Mohamed M, Schmitz-Eiberger N, Keutgen, Noga G. Comparative drought postponing and tolerance potentials of two tepary bean lines in relation to seed yield. | Afr Crop Sci J. 2005; 13: 49-60. |
| 63. | Muchow RC. Phenology, seed yield and water use of grain legumes grown under different soil water regimes in a semi arid tropical environment. | Field Crops Res. 1985; 11: 81-97. |
| 64. | Nageswara Rao RC, Singh S, Sivakumar MVK, Srivastava KL, Williams JH. Effects of water deficit at different growth phases of peanut: 1. Yield responses. | Agron J. 1985; 77: 782-786. |
| 65. | Nageswara Rao RC, Williams JH, Sivakumar MVK, Wadia KDR. Effect of water deficit at different growth phases of peanut: 2. Response to drought during preflowering phase. | Agron J. 1988; 80: 431-438. |
| 66. | Nam NH, Chauhan YS, Johansen E. Effects of timing of drought stress o growth and grain yield of extra-short duration of pigeonpea lines. | J Agr Sci. 2001; 136: 179-189. |
| 67. | Nautiyal PC, Ravindra V, Joshi YC. Dry matter partitioning and water use efficiency under water-deficit during various growth stages in groundnut. | Indian J Plant Physiol. 2002; 7: 135-139. |
| 68. | Ngwako S, Balole TV, Malambane G. The effect of irrigation and planting date on the growth and yield of Bambara groundnut landraces. | Int J Agr Crop Sci. 2013; 6: 116-120. |
| 69. | Nielsen DC, Nelson NO. Black bean sensitivity to water stress at various growth stages. | Crop Sci. 1998; 38: 422-427. |
| 70. | Oweis T, Hachum A, Pala M. Faba bean productivity under rainfed and supplemental irrigation in northern Syria. | Agric Water Manage. 2005; 73: 57-72. |
| 71. | Oya T, Nepomuceno AL, Neumaier N, Farias JRB, Tobita S, et al. Drought tolerance characteristics of Brazilian soybean cultivars – Evaluation and characterization of drought tolerance of various Brazilian soybean cultivars in the field. | Plant Prod. Sci. 2004; 7: 129-137. |
| 72. | Pahalwan DK, Tripathi RS. Irrigation scheduling based on evaporation and crop water requirement for summer peanuts. | Peanut Sci. 1984; 11: 4-6. |
| 73. | Pandey RK, Herrera WAT, Pendleton JW. Drought response of grain legumes under irrigation gradient: 1. Yield and yield components. | Agron J. 1984; 76: 549-553. |
| 74. | Pannu RK, Singh DP. Effect of irrigation on water use, water-use efficiency, growth and yield of mungbean. | Field Crop Res. 1993; 31: 87-100. |
| 75. | Patil BP, Gangvane SB. Effects of water stress imposed at various growth stages on yield of groundnut and sunflower. | J Maharashtra Agric Univ. 1990; 15: 322-324. |
| 76. | Pilbeam CJ, Akatse JK, Hebblethwaite PB, Wright SD. Yield production in two contrasting forms of spring-sown faba bean in relation to water supply. | Field Crops Res. 1992; 29: 273-287. |
| 77. | Plaut Z, Ben-Hur M. Irrigation management of peanut with a moving sprinkler system: Runoff, yield and water use efficiency. | Agron J. 2005; 97: 1202-1209. |
| 78. | Peña-Cabriales JJ, Castellanos JZ. Effects of water stress on N_2_ fixation and grain yield of *Phaseolus vulgaris* L. | Plant Soil. 1993; 152: 151-155. |
| 79. | Prabowo A, Prastowo B, Wright GC. Growth, yield and soil water extraction of irrigated and dryland peanuts in south Sulawesi, Indonesia. | Irrig Sci. 1990; 11: 63-68. |
| 80. | Puangbut D, Jogloy S, Vorasoot N, Akkasaeng C, Kesmala T, et al. Variability in yield responses of peanut (*Arachis hypogaea* L.) genotypes under early season drought. | Asian J Plant Sci. 2009; 8: 254-264. |
| 81. | Puangbut D, Jogloy S, Vorasoot N, Akkasaeng C, Patanothai A. Association of transpiration efficiency with N_2_ fixation of peanut under early season drought. | Int J Plant Prod. 2011; 5: 381-394. |
| 82. | Rajin Anwar M, McKenzie BA, Hill GD. The effect of irrigation and sowing date on crop yield and yield components of Kabuli chickpea (*Cicer arietinum* L.) in a cool-temperate subhumid climate. | J Agr Sci. 2003; 141: 259-271. |
| 83. | Rahmiana AA, Taufik A, Yusnawan E. Pod yield and kernel quality of peanut grown under two different irrigations and two harvest times. | Indones J Agr. 2009; 2: 103-109 |
| 84. | Rajin Anwar M, McKenzie BA, Hill GD. Water use efficiency and the effect of water deficit on crop growth and yield of Kabuli chickpea (*Cicer arietinum* L.) in a cool-temperate subhumid climate. | J Agr Sci. 2003; 141: 285-301. |
| 85. | Ramirez-Vallejo P, Kelly JD. Traits related to drought resistance in common bean. | Euphytica 1998; 99: 127-136. |
| 86. | Ratnakumar P, Vadez V. Groundnut (*Arachis hypogaea*) genotypes tolerant to intermittent drought maintain a high harvest index and have small leaf canopy under stress. | Funct Plant Biol. 2011; 38: 1016-1023. |
| 87. | Reddy CR, Reddy SR. Scheduling irrigation for peanuts with variable amounts of water. | Agric Water Manage. 1993; 23: 1-9. |
| 88. | Rosales-Serna R, Ramirez-Vallejo P, Acosta-Gallegos JA, Castillo-Gonzàlez F, Kelly JD. Grain yield and drought tolerance of common bean under field conditions. | Agrociencia 2000; 34: 153-165. |
| 89. | Sadeghipour O. Effect of withholding irrigation at different growth stages on yield and yield components of mung bean (*Vigna radiata* L. Wilczek) varieties. | Am-Eurasian J Agric Environ Sci. 2008; 4: 590-594. |
| 90. | Selvaraju R. Effect of irrigation on phenology, growth and yield of cowpea (*Vigna unguiculata*). | Indian J Agron. 1999; 44: 377-381. |
| 91. | Samarah N, Mullen R, Cianzio S. Size distribution and mineral nutrients of soybean seeds in response to drought stress. | J Plant Nutr. 2004; 27: 815-835. |
| 92. | Senthong C, Pandey RK. Response of five legume crops to an irrigation gradient imposed during reproductive growth. | Agron J. 1989; 81: 680-686. |
| 93. | Shouse P, Dasberg S, Jury WA, Stolzy LH. Water deficits effect on water potential, yield and water use of cowpeas. | Agron J. 1981; 73: 333-336. |
| 94. | Simsek M, Nomlekcioglu N, Ozturk I. The effects of regulated deficit irrigation on yield and some yield components of common bean (*Phaseolus vulgaris* L.) under semi-arid conditions. | Afr J Biotechnol. 2011; 10: 4057-4064. |
| 95. | Sincik M, Candogan BN, Demirtas C, Büyükcangaz H, Yazgan S, et al. Deficit irrigation of soybean (*Glycine max* (L.) Merr.) in a sub-humid climate. | J Agron Crop Sci. 2008; 194: 200-204. |
| 96. | Singh B, Sandhu BS, Khera KL, Aujla TS. Groundnut response to irrigation and sowing time on a deep loamy sand in a subtropical monsoon region. | Field Crop Res. 1986; 13: 355-366. |
| 97. | Singh DP, Singh P, Sharma HC, Turner NC. Influence of water deficit on water relations, canopy gas exchange, and yield of chickpea (*Cicer arietinum*). | Field Crop Res. 1987; 16: 231-241. |
| 98. | Snyder RL, Carlson RE, Shaw RH. Yield of indeterminate soybeans in response to multiple periods of soil-water stress during reproduction. | Agron J. 1982; 74: 855-859. |
| 99. | Songsri P, Jogloy S, Kesmala T, Vorasoot N, Akkasaeng C, et al. Heretability of drought resistance traits and correlation of drought resistance and agronomic traits in peanut. | Crop Sci. 2008; 48: 2245-2253. |
| 100. | Stansel JR, Pallas Jr JE. Yield and quality response of florunner peanut to applied drought at several growth stages. | Peanut Sci. 1985; 12: 64-70. |
| 101. | Stegman EC, Schatz BJ, Garder JC. Yield sensitivities of short season soybeans to irrigation management. | Irrig Sci. 1990; 11: 111-119. |
| 102. | Szilagyi L. Influence of drought on seed yield components in common bean. | Bulg J Plant Physiol. 2003; Special Issue 320-330. |
| 103. | Tabrizi MS, Parsinejad M, Babazadeh H. Efficacy of partial root drying technique for optimizing soybean crop production in semi-arid regions. | Irrig Drain. 2012; 61: 80-88. |
| 104. | Thiyagarajan G, Ranghaswami MV, Rajakumar D, Kumaraperumal R. Deficit irrigation effects on groundnut (*Arachis hypogaea* L.) with micro sprinklers. | Madras Agric J. 2010; 97: 40-42. |
| 105. | Thomas, Fukai S. Growth and yield response of barley and chickpea to water stress under three environments in southeast Queensland. 1 Light interception, crop growth and grain yield. | Aust J Agric Res 1995; 46, 17-33. |
| 106. | Thomas, Robertson MJ, Fukai S, Peoples MB. The effect of timing and severity of water deficit on growth, development and yield accumulation and nitrogen fixation of mungbean. | Field Crop Res. 2004; 86: 67-80. |
| 107. | Turk KJ, Hall AE, Asbell CW. Drought adaptation of cowpea. 1 Influence of drought on seed yield. | Agron J. 1980; 72: 413-420. |
| 108. | Ucar Y, Kadayifci A, Yilmaz HI, Tuylu GI, Yardimci N. The effect of deficit irrigation on the grain yield of dry bean (*Phaseolus vulgaris* L.) in semiarid regions. | Span J Agric Res. 2009; 7: 474-485. |
| 109. | White JW, Castillo JA, Ehleringer JR, Garcia-C JA, Singh SP. Relations of carbon isotope discrimination and other physiological traits to yield in common bean (*Phaseolus vulgaris*) under rainfed conditions. | J Agr Sci. 1994; 122: 275-284. |
| 110. | Wright GC, Hubick KT, Farquhar GD. Physiological analysis of peanut cultivar response to timing and duration of drought stress. | Aust J Agric Res. 1991; 42: 453-470. |
| 111. | Zaman-Allah M, Jenkinson DM, Vadez V. A conservative pattern of water use, rather than deep or profuse rooting, is critical for the terminal drought tolerance of chickpea. | J Exp Bot. 2011; 62: 4239-4252. |
